# Late-onset Niemann–Pick disease type C overlapping with frontotemporal dementia syndromes: a case report

**DOI:** 10.1007/s00702-019-02058-0

**Published:** 2019-09-10

**Authors:** Nóra Balázs, Dániel Milanovich, Csilla Hornyák, Dániel Bereczki, Tibor Kovács

**Affiliations:** Department of Neurology, Semmelweis University, Balassa utca 6, Budapest, 1083 Hungary

**Keywords:** Niemann–Pick disease type C, Vertical supranuclear gaze palsy, Frontotemporal dementia, Corticobasal syndrome, Progressive supranuclear palsy

## Abstract

The diagnosis of adult-onset Niemann–Pick disease type C (NPC) could be difficult because its primary symptoms [dementia and vertical supranuclear gaze palsy (VSGP)] are mainly seen in neurodegenerative dementias and progressive supranuclear palsy (PSP). Our patient with dementia and asymmetric parkinsonism resembled corticobasal syndrome and after the appearance of VSGP, the criteria of PSP were fulfilled too. Cerebellar symptoms appeared late during the course of the disease, leading to the diagnosis of NPC at the age of 59 years.

## Introduction

Niemann–Pick disease type C (NPC) is a rare genetic disorder belonging to the lysosomal storage diseases. It is mainly diagnosed in children, while adult-onset forms of the disease are even rarer. Diagnosis of late-onset NPC is complicated by the heterogeneous symptomatology of the disease, especially in adults, where its neuropsychiatric consequences (mainly psychosis and dementia) are frequent symptoms in degenerative dementias and the red flag sign of NPC, the vertical supranuclear gaze palsy (VSGP), is usually seen in patients with progressive supranuclear palsy (PSP) (Höglinger et al. 2017). In this case report, we present our patient diagnosed with NPC at the unusually late age of 59 years with symptomatology resembling PSP and corticobasal syndrome (CBS) (Armstrong et al. 2013).

## Case report

The mother of our male patient died at the age of 55 years and three of his seven siblings were affected, two of them died at the ages of 65 and 50 years, without diagnosis. Consanguinity was found in the patient pedigree.

**Fig. 1:**
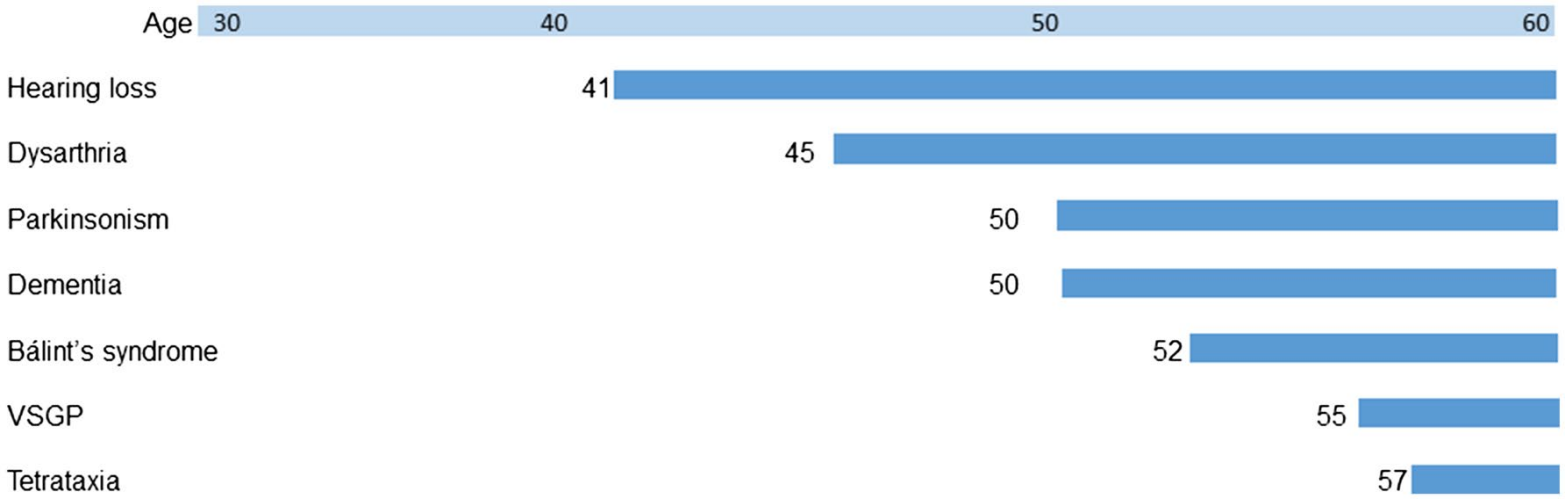
Appearance of symptoms in our patient

**Fig. 2:**
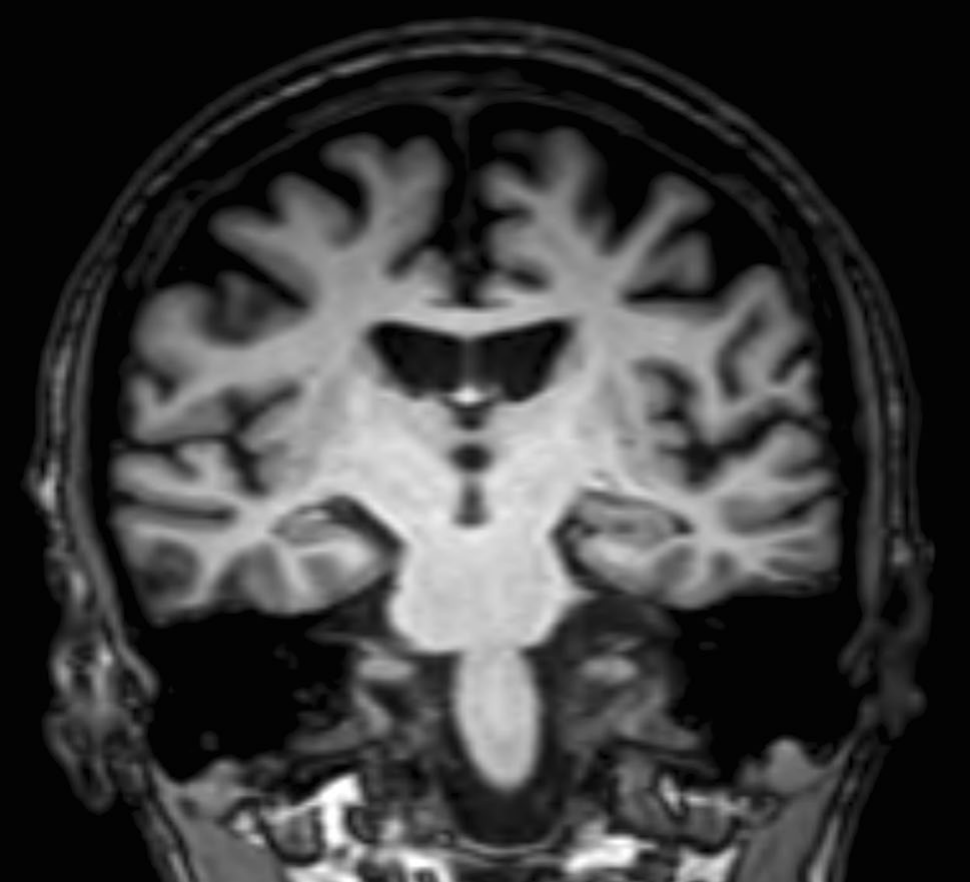
Mild atrophy on coronal T1-weighted MR image of the patient at the age of 56 years

The first complaints developed in his fifth decade of life (Fig. 1). He used hearing aids from the age of 41 years and his speech became slurred at the age of 45 years. During his first neurological examination at the age of 50 years, dysarthria, unsteady gait, frontal release signs, mild prefrontal symptoms (decreased verbal fluency, concretization, mild perseveration), left-sided dominant parkinsonism (without response to levodopa) with early postural instability were found with mild atrophy on brain MRI (Fig. 2). Orofacial apraxia and Bálint’s syndrome (optic ataxia, ocular apraxia and simultaneous visual agnosia) developed 2 years later and the patient was diagnosed with CBS (Armstrong et al. 2013).

During his follow-up examinations, repeated brain magnetic resonance imaging showed generalized cerebral atrophy without disease-specific pattern and progression. Genetic tests for tau and prion protein genes were negative.

VSGP appeared at the age of 55 years, after at least 10 years from symptom onset, shifting the diagnosis to PSP, without suggestive features of PSP other than levodopa-resistance (Höglinger et al. 2017). 2 years later, cerebellar symptoms (mild tetraataxia) appeared, raising the suspicion (in spite of the age of the patient) of NPC, which was proven by elevated blood plasma oxysterol (0.196 ng/ml) and lyso-sphingomyelin-509 (1.0 ng/ml). Genetic testing of the patient showed the mutations of c.2861C > T (S954L) and c.3019C > G (P1007A) in the NPC1 gene (probably with compound heterozygosity; segregation analysis was not possible, parents of the patient died), both mutations are already known to be disease causing (Greer et al. 1999). These results confirmed the diagnosis of NPC and miglustat therapy was initiated. Abdominal ultrasound was performed for screening of visceral manifestations and hepato-splenomegaly was found.

## Discussion

Niemann–Pick (NP) disease is a rare neurovisceral disorder with autosomal recessive inheritance. NP has two distinct forms. The first is due to complete or partial deficiency of acid sphingomyelinase, resulting from mutations in the SMPD1 gene with the clinical phenotypes of NP type A and B. NPC is characterized by unique abnormalities of intracellular transport of endocytosed cholesterol with sequestration of unesterified cholesterol in lysosomes and late endosomes, but the function of the affected proteins (NPC1 and NPC2, encoded by NPC1 and NPC2 genes, respectively) are not precisely elucidated (Ory 2004). Age of onset ranges from the perinatal period until the seventh decade of life and the lifespan of the patients varies between a few days until over 60 years, although the majority of the patients die between 10 and 25 years of age (Tang et al. 2010). The oldest reported patient with NPC was diagnosed at the age of 68 years (Trendelenburg et al. 2006).

The manifestations of NPC are classified in visceral, neurological and psychiatric categories. While visceral manifestations tend to predominate during the perinatal and infantile period, neurological and psychiatric signs are more prominent in juvenile and adult patients. Neurological manifestations of NPC include non-disease-specific and more pathognomonic neurological signs such as VSGP especially when it occurs in combination with other manifestations, e.g., ataxia or splenomegaly (Tang et al. 2010; Walterfang et al. 2006). In case of our patient-in contrast to the usual pattern in NPC (Patterson et al. 2012), VSGP appeared late during the course of the disease and cerebellar symptoms were the last to manifest, leading to the suspicion of NPC.

The symptoms of NPC might overlap with the spectrum of frontotemporal dementias (FTD), as seen in our patient who had dementia with asymmetric parkinsonism as the initial manifestation of NPC.

Based upon the predominant early feature of the disease, FTD can be categorized into three main syndromes: behavioral variant (bvFTD), semantic variant (svPPA) and non-fluent variant of primary progressive aphasia (nfvPPA). Motor neuron signs and parkinsonism are frequent accompanying symptoms (FTD with motor neuron disease (FTD-MND), PSP and CBS are being the corresponding clinical syndromes) (Oeckl et al. 2016). Pick complex is a unifying concept of these overlapping phenotypes, with the usual feature of changing in the main clinical pattern during the course of the disease (Kertesz et al. 2005). Signs and symptoms of our patient fulfill the criteria of PSP (progressive gait disturbance with postural instability, levodopa resistant parkinsonism and VSGP; “probable” PSP, except for the exclusionary criterion of a proven non-tau-related pathogenetic process) (Höglinger et al. 2017) and CBS (progressive asymmetrical rigidity and apraxia; “possible” CBS) too (Armstrong et al. 2013). PSP has to be a sporadic disease, although rare tau gene variants (mutations) may lead to inherited phenocopies of the sporadic disease with a Mendelian trait pattern (Höglinger et al. 2017). Diagnosis of PSP and CBS is difficult due to the lack of specific in vivo biomarkers of these disorders. The accumulation of the phenotype in our patient’s family caused a pseudodominant pattern of this recessive disorder probably because of consanguinity.

Neurological manifestations in combination with frequently asymptomatic visceral symptoms raise the suspicion of NPC. In patients with adolescent/adult-onset form, splenomegaly can only be occasionally detected by routine ultrasound assessments. Score systems aid clinicians to estimate the probability of NPC (such as www.npc-si.com). In case of high likelihood of NPC there are available biochemical and genetic tests to prove the diagnosis. A sensitive and specific biomarker is oxysterol, a cholesterol oxidation product, which level is significantly associated with the age of initial presentation and severity of the disease (Papandreou and Gissen 2016). Lyso-sphingomyelin-509 is a new biomarker with quicker and easier measurement methods (Papandreou and Gissen 2016).

Genetic tests are requested to support suspicious biochemical results. NPC1 gene mutations are present in 95% of cases, while NPC2 mutations in approximately 4%; the remaining patients are biochemically proven cases who do not have identified mutations (Papandreou and Gissen 2016).

Only one study was found in the literature where a limited number of PSP and FTD patients had been screened for NPC1 and NPC2 gene mutations, but no carriers were found in these patient groups (Papandreou and Gissen 2016). However, our case is calling attention to keep in mind the possibility of NPC in adult patients with clinical phenotype resembling PSP, CBS or FTD even in older ages than 40 and 50 years, which are the age limits of PSP and CBS, respectively, in their diagnostic criteria (Höglinger et al. 2017; Armstrong et al. 2013).

## Conclusion

NPC may be under-diagnosed due to its highly heterogeneous clinical presentation. The neurological manifestations of NPC can mimic FTD syndromes leading to significant delays in the diagnosis especially in dementia and movement disorders units, where vertical gaze palsy is a red flag symptom for PSP. In case of familial aggregation of the phenotype and presence of otherwise inexplicable visceral symptoms, clinicians should think about late-onset NPC, where treatment with miglustat is able to slow the progression of the disease (Papandreou and Gissen 2016).
